# Balance in Transition: Unraveling the Link Between Menopause and Vertigo

**DOI:** 10.7759/cureus.59277

**Published:** 2024-04-29

**Authors:** Melissa Castillo-Bustamante, Neşe Çelebisoy, Luis G Echavarria, Isabela Franco, Santiago Valencia, Sara Gonzalez, Alejandro García

**Affiliations:** 1 Otoneurology, Centro de Vértigo y Mareo, Mexico City, MEX; 2 Medicine, Universidad Pontificia Bolivariana, Medellín, COL; 3 Neurology, Ege University Medical School, Izmir, TUR; 4 Obstetrics and Gynecology, Clinica Universitaria Bolivariana, Medellín, COL; 5 Otolaryngology, Universidad Pontificia Bolivariana, Medellín, COL; 6 Otolaryngology, Universidad Pontificia Bolivariana, Medellin, COL; 7 Ear, Nose and Throat (ENT), The University of Iowa, Iowa, USA

**Keywords:** obstetrics & gynecology, vertigo, sex hormones, menopause, vestibular disorders

## Abstract

The onset of menopause, marked by hormonal fluctuations and a decline in estrogen levels, is suggested to be linked to increased susceptibility to vestibular disturbances. Estrogen, beyond its established association with reproductive physiology, plays modulatory roles in various physiological systems, including neurosensory function. The vestibular system, crucial for balance and spatial orientation, is influenced by hormonal changes during menopause, potentially contributing to the emergence of vertigo symptoms. This interplay between hormones and the vestibular system is a burgeoning area of research with clinical implications, offering insights into novel diagnostic and therapeutic approaches for managing postmenopausal women with vestibular disorders. The article reviews current scientific literature, delves into the hormonal intricacies of menopause, and investigates potential mechanisms underlying the connection between hormonal fluctuations and vertigo symptoms.

## Introduction and background

Vertigo is a complex and multifaceted phenomenon that significantly impacts the quality of life for many individuals [1]. While the etiology of vertigo is diverse, emerging evidence suggests a potential link between the onset of menopause and an increased susceptibility to vestibular disturbances [2]. Menopause, a natural biological process marking the end of a woman's reproductive years, is characterized by hormonal fluctuations, most notably a decline in estrogen levels [3]. This pivotal period of hormonal transition is known to influence various physiological systems, including the intricate mechanisms governing balance within the inner ear [4]. Beyond its well-established association with reproductive physiology, estrogen is increasingly recognized for its modulatory roles in various physiological systems, including those governing neurosensory function. One intriguing dimension of this interplay is the emergence of vertigo, a distressing vestibular symptom characterized by a distorted sense of motion and spatial orientation [2-4]. The vestibular system, comprising the inner ear structures and complex neural networks, plays a central role in maintaining equilibrium and spatial orientation [5]. Recent investigations have unveiled a potential link between the hormonal fluctuations accompanying menopause and the manifestation of vertigo symptoms [2,6]. Understanding the nuanced relationship between hormonal dynamics and vestibular function during this pivotal life stage is not only of academic interest but also holds substantial clinical relevance [7].

The complex interplay between hormones and the vestibular system is an intriguing avenue of research that has gained momentum in recent years [2]. Understanding the relationship between menopause and vertigo holds significant clinical implications, as it may shed light on novel diagnostic and therapeutic approaches for managing vestibular disorders in postmenopausal women [2]. This article reviews the current scientific literature, explores the hormonal intricacies of menopause, and investigates the potential mechanisms through which hormonal fluctuations may contribute to the development or exacerbation of vertigo symptoms.

## Review

Methods

Literature Search Strategy

Our narrative review was conducted systematically to identify relevant studies investigating the association between vertigo and menopause. A thorough search of electronic databases, including PubMed, MEDLINE, Embase, and Web of Science, was performed. The search strategy employed a combination of keywords such as "menopause," "hormonal changes," "vertigo," and "vestibular disorders." The search included articles from 1960 to 2023.

The inclusion criteria were articles reporting vertigo during menopause, including the following types of studies: case reports, retrospective chart reviews, cross-sectional studies, case-control cohorts, and systematic reviews. Additionally, articles reporting Meniere’s disease, vestibular neuritis, benign paroxysmal positional vertigo (BPPV), and other presentations of peripheral vertigo during menopause were included. Editorials, narrative reviews, scope reviews, letters to the editor, comments, and abstracts were excluded. Articles not focused on vertigo during menopause and those focused on vertigo during perimenopause, hormone replacement therapy, and chemically induced menopause were also excluded. Reports of vertigo during menopause associated with inner ear malformations, arteriovenous malformations, and central nervous system diseases were also excluded. In total, 92 articles were found, of which 7 articles were included in this review. All articles selected were cross-checked by the authors. Data extraction was performed systematically, and relevant information was recorded, including study design, sample size, participant characteristics, hormonal measures, vertigo assessment methods, and key findings. The quality of the included observational studies was independently assessed by two reviewers, focusing on relevance to the topic of vertigo in menopause and methodological rigor. The assessment was conducted using the Newcastle-Ottawa Scale (NOS), which evaluates study quality based on the selection of study groups, comparability of groups, and ascertainment of outcomes. Each study received a score reflective of its methodological robustness, with higher scores indicating higher quality. The narrative synthesis approach was chosen to present the findings coherently and comprehensively. Themes and patterns emerging from the literature were identified, and the synthesis process was iterative, allowing for the integration of diverse study designs and methodologies.

Review

Menopause represents a pivotal and natural phase in a woman's life, denoting the permanent cessation of menstrual cycles and the culmination of the reproductive years [8,9]. This intricate biological process is tightly linked to the gradual decline of ovarian function, marked by the depletion of ovarian follicles and a subsequent reduction in the production of sex hormones, predominantly estrogen and progesterone [8,9]. The average age of menopause is approximately 51 years, although the timing can vary widely among individuals [8,9]. Beyond its fundamental biological underpinnings, menopause manifests a spectrum of physiological, hormonal, and clinical changes, giving rise to a myriad of symptoms and implications that extend well beyond the reproductive system [10,11]. Menopause, often preceded by a stage known as perimenopause, introduces a complex interplay of hormonal fluctuations, impacting diverse organ systems and engendering a range of symptoms such as vasomotor symptoms (e.g., hot flashes, night sweats), genitourinary changes, and alterations in bone density [10-13]. While traditionally associated with reproductive changes, emerging evidence suggests a potential link between menopause and vestibular dysfunction, manifesting as symptoms of vertigo [12,13].

Estrogen, a key reproductive hormone, exhibits a complex interplay with the central nervous system, influencing various physiological functions [14]. During menopause, a woman experiences a significant decline in estrogen levels, which may contribute to alterations in neuronal function. Estrogen receptors are present in the vestibular system, and their modulation can impact the processing of spatial orientation and balance [14,15]. The intricate dance between estrogen and the CNS involves the modulation of neurotransmitters, neural plasticity, and neuroprotection [14,15]. As estrogen levels decline, these processes may be disrupted, potentially impacting the delicate equilibrium of the vestibular system [16].

The vestibular system, comprising the inner ear and associated neural pathways, plays a crucial role in maintaining balance and spatial orientation [17,18]. Estrogen receptors within the vestibular system suggest a direct hormonal influence on its function [17-19]. Reduced estrogen levels during menopause may lead to changes in vestibular sensitivity and response, potentially contributing to the onset of vertigo [2].

Vestibular facts and types of vertigo during menopause

The interaction between the endocrine and vestibular systems is still poorly documented [2]. However, some studies show clear links between both systems [2,20]. During menopause, patients experience hormonal lability, mainly a reduction in estrogen levels [21-24]. Estrogens, besides their reproductive functions, are essential in organs such as bones, the cardiovascular system, and the central nervous system, where they specifically act on the postural balance system by integrating signals from the vestibular, visual, and proprioceptive systems [21-24].

During menopause, patients commonly report various types of vertigo, including hormonally mediated vertigo, BPPV, vestibular migraine, and Meniere's disease [20-23]. The lifetime prevalence of these vertigo types is estimated to be in the range of 23-30%, with ongoing research to further refine these figures [20-23]. These conditions are believed to share a common pathophysiological mechanism characterized by reduced levels of serum estrogen (E2) in menopausal patients [22]. Women may experience a correlation between the severity of vertigo episodes and specific phases of the menstrual cycle, highlighting the influence of hormonal variations on vestibular function [2,22]. Hormonal fluctuations may be implicated in an increased tendency to develop BPPV or vestibular migraine [2,22]. Although this specific type of vertigo is acknowledged, additional studies are imperative to provide a comprehensive clinical description [2,22]. Meanwhile, various other common types of vertigo are outlined below [2,22].

Benign Paroxysmal Positional Vertigo (BPPV)

BPPV, a condition characterized by brief episodes of vertigo triggered by changes in head position, is another reported subtype during menopause [20,21]. While the exact mechanisms linking menopause and BPPV are not fully elucidated, hormonal fluctuations may influence the composition of inner ear fluids, contributing to the dislodgment of otolith particles and subsequent vertigo episodes [6].

BPPV is caused by the detachment of otoconia from the utricle toward the semicircular canals, causing short, recurrent episodes of vertigo generally triggered by positional changes [21]. This type of vertigo, with 50-70% of cases having an idiopathic etiology, can also have identifiable causes such as surgery, traumatic brain injury, Meniere's disease, and vestibular neuritis [23]. Associated risk factors include hypertension, diabetes mellitus, dyslipidemia, autoimmune thyroiditis, osteoporosis, and vitamin D deficiency [21-23]. This type of vertigo significantly affects 420 million people worldwide, with a prevalence of 10% [24].

The pathophysiology of vertigo episodes reported by patients during menopause may be attributed to a decrease in estrogen levels [20]. Estrogens play a pivotal role in the metabolism of otoconia, which are biocrystals composed of calcium carbonate and proteins such as otoconin 90 [25]. The decline in estrogen concentration during menopause disrupts the autophagic process of these biocrystals, leading to the formation of larger otoconia and partially explaining the pathophysiology of vertigo observed in menopausal patients [25,26].

As part of the natural aging process, the decline in estrogen levels may lead to the degeneration and dislocation of otoconia [20-25]. Beyond its impact on the cochlea, estrogen and its receptors exert effects on the inner ear, potentially influencing endolymph ionic and anionic homeostasis by regulating the expression of ion channels and pumps [20-25]. The balance of endolymphatic ions and anions plays a crucial role in otoconia/otolith formation [20-25]. This regulatory influence on ion homeostasis could be an additional mechanism underlying the impact of estrogen on the maintenance and anchoring of otoconia [20-25]. It is plausible that a sudden decrease or increase in estrogen levels could disrupt anion/ion homeostasis, concurrently affecting neurosensory function and contributing to the higher prevalence of BPPV observed in older women.

Furthermore, there is an alteration in otoconia metabolism associated with the reduction in estrogen receptors, primarily the alpha receptor, secondary to decreased serum estrogen levels [24]. Consequently, estradiol and vitamin D deficiency have been proposed as significant risk factors for BPPV in postmenopausal women [6,25]. This complex interplay between hormonal changes and otoconia dynamics provides valuable insights into the mechanisms underlying vertigo during the menopausal transition [6,25].

Vestibular Migraine

Vestibular migraine is defined as recurrent episodes of vertigo in a patient with a history of migraine or clinical findings of migraine, accompanied by symptoms such as nausea, vomiting, and a sensation of movement [27]. This type of vertigo affects women more than men at a ratio of 5:1.5 and has a peak diagnosis age of 42 years [28]. Vestibular migraine has an incidence of 1% [27,28].

Menopause can unmask or exacerbate vestibular migraine, a subtype of migraine characterized by vertigo as a prominent symptom [27,28]. The hormonal shifts during menopause may trigger or worsen vestibular migraine attacks in susceptible individuals [26-28]. The overlap between hormonal changes and migraine pathophysiology underscores the importance of considering vestibular migraine in the differential diagnosis of vestibular attacks during the menopausal period [26-28].

In vestibular migraine, the critical serum estrogen concentration is known to be 45-50 pg/ml, with low levels leading to the activation of the trigeminovascular system [28]. Ovarian steroids, including estrogen and progesterone, exert profound influences on the CNS. They are not only synthesized within the CNS but also transported and metabolized in the brain [16]. Ovarian neurosteroid receptors are widely distributed across the cerebral cortex, subcortex, and cerebellum [29]. Estrogen and progesterone play pivotal roles in modulating various neurotransmitter systems associated with migraine activation, including the serotonergic, glutamatergic, GABAergic, noradrenergic, and opioid systems [29]. Estrogen primarily facilitates the glutamatergic and serotonergic systems, with additional facilitatory and inhibitory effects on the opiate and noradrenergic systems [30]. Conversely, progesterone and its metabolites activate GABAergic systems and modulate the actions of estrogen on the central nervous system [30]. These neurosteroid effects also influence the pain processing network in the brainstem, a crucial component of the migraine activation pathway [30]. Generally, estrogen induces neuronal hyper-responsiveness, while progesterone induces neuronal hypo-responsiveness by modulating estrogen action in the trigeminal nucleus caudalis (TNC) [30].

There are four potential pathogenetic mechanisms explaining the vestibular involvement in the migraine pathway [28,31]. Firstly, neurotransmitter systems implicated in migraine pathogenesis also modulate the activity of the vestibular nucleus, establishing a bidirectional influence [28,31]. Secondly, cortical spreading depression may play a role when cortical areas processing vestibular information are engaged, explaining the temporal characteristics of vestibular symptoms [28,31]. Thirdly, the peripheral vestibular system, marked by a high prevalence of abnormal vestibular function tests, may contribute significantly to the pathogenetic mechanism of vestibular migraine, involving potent vasodilators such as substance P, neurokinin A, and calcitonin gene-related peptide [28,31]. Lastly, genetically determined ion channel defects may be another mechanism involving both central and peripheral vestibular systems, contributing to the pathogenesis of vestibular migraine [28,31].

Meniere's Disease

Meniere's disease is a chronic and disabling inner ear disorder characterized by recurrent and spontaneous episodes of vertigo, often accompanied by sensorineural hearing loss, aural fullness, and pulsatile tinnitus [32]. The hallmark of Meniere's disease is the unpredictability of its attacks, which can last anywhere from 20 minutes to several hours and may occur intermittently over an extended period [32]. Psychopathological alterations, particularly anxiety, and socioeconomic factors such as poor support from close individuals and a low socioeconomic level, contribute to the risk of Meniere's disease. This type of vertigo primarily occurs in women aged 40-60 and is associated with the physiopathology of endolymphatic hydrops [33].

Menopausal patients with Meniere's disease exhibit lower estradiol levels, potentially influencing neuronal plasticity, the metabolic levels of neurotransmitters, and consequently, the neuronal conduction time within the audio-vestibular system [34].

Low estrogen levels could be considered as additional underlying factors that may trigger vestibular attacks; however, further studies are needed to gain a better understanding [22].

Facts and challenges

The psychological impact of menopausal symptoms, coupled with hormonal fluctuations, may contribute to anxiety-related vertigo [35]. Women undergoing menopause often report heightened anxiety levels, and this emotional stress can exacerbate or even trigger episodes of vertigo [35]. The bidirectional relationship between anxiety and vertigo emphasizes the importance of addressing both aspects in a comprehensive treatment approach [36].

Vertigo during menopause presents a diagnostic challenge due to the multifactorial nature of its etiology [20]. The intersection of hormonal fluctuations, aging-related physiological changes, and potential comorbidities complicates the accurate identification and categorization of the underlying causes [20]. The episodic and unpredictable nature of vertigo during menopause can substantially diminish the overall quality of life for affected individuals [6,20]. The physiological and psychological consequences of recurrent vertigo episodes may lead to heightened anxiety, fear, and lifestyle limitations, influencing both physical and emotional well-being [6,20].

Vertigo in the menopausal context often coexists with a myriad of other symptoms, such as vasomotor symptoms and mood disturbances [35]. This symptomatic overlap poses challenges in isolating and understanding the specific contribution of vertigo to the overall clinical presentation [35]. The similarity in symptomatology between vestibular migraine and BPPV raises the risk of misdiagnosis in some patients [37]. A precise and accurate differential diagnosis is crucial for effective management [37].

The hormonal milieu, particularly the decline in estrogen levels, is implicated in the pathogenesis of vestibular symptoms during the menopausal period [20]. Managing these hormonal fluctuations and delineating their intricate influence on the vestibular system present challenges in developing targeted therapeutic interventions [20]. Constructing an efficacious treatment strategy for these patients is intricate. Hormone replacement therapy (HRT) and other interventions may be considered, but their optimal balance must be carefully determined to alleviate symptoms without exacerbating concurrent menopausal manifestations [38].

Regardless of the type of vertigo experienced by patients during menopause, its management is crucial, and hormone replacement therapy can be administered using phytoestrogens, raloxifene (a selective estrogen receptor modulator), or combined estrogen and progestogen or estrogen-alone therapy [38]. This treatment not only prevents morphological changes in otoconia and future vertigo episodes in patients with BPPV [6,24]. Besides hormone replacement, improving patients' condition requires vitamin D supplementation since this vitamin is crucial for otoconia formation, maintaining normal calcium ion concentrations, and preventing labyrinth mineralization [39]. During menopause, vitamin D levels decrease, putting patients at risk of vertigo recurrence [40].

Beyond its impact on the vestibular system, menopause affects broader aspects of health, including cardiovascular health and mental well-being [41]. A comprehensive understanding of these interactions is imperative for a holistic approach to managing the multifaceted challenges associated with menopause [41].

While reviewing the relationship between vertigo and menopause, it is essential to acknowledge certain limitations inherent in the available literature [41]. These limitations may include the heterogeneity of study designs, limited randomized controlled trials, diversity in menopausal definitions, limited longitudinal data, and the variability in the characteristics of study participants such as age, comorbidities, and lifestyle factors that may influence the generalizability of findings to the broader population. Long-term data on the progression of vertigo symptoms throughout the menopausal transition are limited. This restricts our understanding of the temporal relationship between hormonal changes and the development of vertigo.

Based on the current state of knowledge and the identified limitations in the review on vertigo and menopause, several proposals for future research and clinical directions can be considered, such as the exploration of hormonal mechanisms, which could provide insights into targeted interventions and potential therapeutic targets. Also, incorporating patient-reported outcomes and quality of life measures to assess the impact of vertigo on individuals during and after menopause is valuable for tailoring interventions that address not only the symptomatology but also its broader effects.

## Conclusions

In summary, the challenges of menopausal vertigo encompass diagnostic intricacies, implications for quality of life, hormonal modulation complexities, risk of misclassification, therapeutic dilemmas, and broader health implications. Addressing these challenges necessitates a rigorous and interdisciplinary approach to provide effective management and enhance the overall well-being of individuals experiencing vertigo during menopause.
